# Influences of Supplementing Selective Members of the Interleukin-6 Cytokine Family on Bovine Oocyte Competency

**DOI:** 10.3390/ani14010044

**Published:** 2023-12-21

**Authors:** Endya McKinley, Savannah L. Speckhart, Jessica A. Keane, Mary A. Oliver, Michelle L. Rhoads, J. Lannett Edwards, Fernando H. Biase, Alan D. Ealy

**Affiliations:** 1 School of Animal Sciences, Virginia Tech, Blacksburg, VA 24061, USA; e.mckinley@conceptionsflorida.com (E.M.); sspeckhart@kumc.edu (S.L.S.); jakeane@vt.edu (J.A.K.); maryalio@vt.edu (M.A.O.); rhoadsm@vt.edu (M.L.R.); fbiase@vt.edu (F.H.B.); 2 Department of Animal Science, The University of Tennessee, Knoxville, TN 37996, USA; jedwards@utk.edu

**Keywords:** cow, oocyte, interleukin, cytokine, in vitro embryo production

## Abstract

**Simple Summary:**

In vitro-produced (IVP) bovine embryos have reduced developmental potential and post-transfer survival. One reason this may occur is because of the lack of growth, differentiation factors, and cytokines that are present within the follicle but absent within oocyte maturation culture systems. This work explored whether supplementing selective members of the interleukin-6 (IL6) cytokine family during in vitro bovine oocyte maturation affects maturation success, cumulus–oocyte complex (COC) gene expression, fertilization success, and embryo development potential. Human recombinant proteins for IL6, IL11, and leukemia inhibitory factor (LIF) were supplemented to COCs during the maturation period, then fertilization and embryo culture commenced without further cytokine supplementation. Several key outcomes were observed. Both LIF and IL11 increased one COC transcript associated with oocyte competency whereas IL6 did not produce this effect. None of the cytokines influenced fertilization success but supplementing COCs with LIF or IL11 increased the ability of these oocytes to generate blastocyst stage embryos. No effect of IL6 supplementation was detected. Collectively, this work provides evidence that supplementing LIF or IL11 during in vitro oocyte maturation complexes improves oocyte competency, but no such effects were detected when IL6 was supplemented during maturation.

**Abstract:**

This work explored whether supplementing selective members of the interleukin-6 (IL6) cytokine family during in vitro bovine oocyte maturation affects maturation success, cumulus–oocyte complex (COC) gene expression, fertilization success, and embryo development potential. Human recombinant proteins for IL6, IL11, and leukemia inhibitory factor (LIF) were supplemented to COCs during the maturation period, then fertilization and embryo culture commenced without further cytokine supplementation. The first study determined that none of these cytokines influenced the rate that oocytes achieved arrest at meiosis II. The second study identified that LIF and IL11 supplementation increases *AREG* transcript abundance. Supplementation with IL6 supplementation did not affect *AREG* abundance but reduced *HAS2* transcript abundance. Several other transcriptional markers of oocyte competency were not affected by any of the cytokines. The third study determined that supplementing these cytokines during maturation did not influence fertilization success, but either LIF or IL11 supplementation increased blastocyst development. No effect of IL6 supplementation on subsequent blastocyst development was detected. The fourth experiment explored whether each cytokine treatment affects the post-thaw survivability of cryopreserved IVP blastocysts. None of the cytokines supplemented during oocyte maturation produced any positive effects on post-thaw blastocyst re-expansion and hatching. In conclusion, these outcomes implicate IL11 and LIF as potentially useful supplements for improving bovine oocyte competency.

## 1. Introduction

Oocyte quality is of paramount importance for cattle and many other mammalian species [1,2]. Adverse environmental and physiological events that occur prior to ovulation can have severe detrimental effects on subsequent fertilization success, embryo developmental potential, and pregnancy success [3,4,5]. This issue with oocyte quality is also concerning when using in vitro embryo production schemes in cattle, where oocytes are harvested from live cows through a transvaginal ovum pickup (OPU) scheme or are collected from abattoir ovaries. Oocytes contained within a mass of cumulus cells, called a cumulus oocyte complex (COC), are matured in vitro then fertilized, and embryos are cultured to the blastocyst stage before transfer to surrogate cattle either before or after cryopreservation [6]. The most recent survey determined that >1.5 million in vitro-produced (IVP) bovine embryos were transferred worldwide in 2021 [7]. There has been a constant upward trend in using IVP bovine embryos in the past decade [7], and all indications are that the use of these embryo will continue to increase. There are two major limitations with this process: one is that the efficiency of in vitro embryo development is low, where only 20% to 40% of oocytes will yield transferable embryos, and a second is that reduced post-transfer competency has been observed in IVP embryos [6,8]. 

One topic of special interest for our laboratory is to better understand how to properly manage the oocyte during in vitro maturation (IVM) so that a greater portion of oocytes can produce transferable embryos and these embryos will be better able to sustain a pregnancy. There is ample evidence that poor oocyte quality is a primary factor contributing to poor in vitro embryo development [9,10] and that poor oocyte quality contributes to poor post-transfer pregnancy success [3,11]. Most IVM systems contain bioactive molecules that will promote oocyte maturation. This work focused on determining whether supplementing selective members of the interleukin-6 (IL6) cytokine family will promote bovine oocyte maturation in ways that result in greater embryo yields. Leukemia inhibitory factor (LIF) has been the best studied member of this family, where supplementation during IVM improved nuclear maturation and increased post-fertilization cleavage rates, blastocyst total cell numbers, and hatching rate [12,13]. Bovine oocyte supplementation with LIF also improved subsequent cryosurvival of oocytes [13]. Other studies where LIF was co-supplemented with fibroblast growth factor 2 (FGF2) and insulin-like growth factor 1 (IGF1) during IVM identified increases in transzonal projection numbers, nuclear maturation, blastocyst development, and blastocyst cryosurvival [14]. There also is evidence suggesting that IL6 and at least one other cytokine family member, termed interleukin 11 (IL11), may mediate bovine oocyte maturation. One supplementation study found that providing IL6 or IL11 during IVM increased the expression of several microRNAs that improve bovine oocyte maturation [13]. Another study suggested that IL6 may participate in mediating the timing of meiosis completion in heat-stress cattle [15]. 

This work was completed to provide a definitive assessment of the comparable and distinct activities of IL6, IL11, and LIF during bovine oocyte maturation. The objective of this study was to determine if supplementing IL6, IL11, or LIF during IVM improves nuclear maturation, COC gene expression, and subsequent cleavage, blastocyst formation, and post-thaw viability of blastocysts.

## 2. Materials and Methods

No animals were used for this work. All studies were completed using abattoir-derived materials (Brown Packing Co., Gaffney, SC, USA) that followed humane slaughter practices according to USDA guidelines. Reagents were purchased from ThermoFisher Chemical Company (Waltham, MA, USA), unless otherwise specified.

### 2.1. In Vitro Maturation 

Maturation procedures were completed as previously described [16]. In brief, cumulus–oocyte complexes (COCs) were harvested from ovaries purchased from Brown Packing Company (Gaffney, SC, USA) and incubated overnight for 22–24 h at 38.5 °C in 5% [*v/v*] CO_2_ in humidified air in groups of 25–35 COCs per 500 µL TCM-199, containing Earle’s salts supplemented with 10% [*ʋ/v*] fetal bovine serum (FBS; Atlanta Biologicals, Flowery Branch, GA, USA), 200 µg/mL Gentamicin, 1 mM Sodium Pyruvate, 2 mM L-alanyl-L-glutamine (Glutamax), 5 µg/mL Folltropin (AgTech Inc., Manhattan, KS, USA), and 20 ng/mL Estradiol (Sigma-Aldrich, St. Louis, MO, USA). 

### 2.2. Supplementation of IL6, IL11, and LIF

Recombinant human IL6, IL11, and LIF were purchased from R&D Systems (Minneapolis, MN, USA) and reconstituted in TCM-199 (Earle’s salts) containing 1% [*w/v*] bovine serum albumin (BSA; Sigma-Aldrich Inc., St. Louis, MO, USA). Single use aliquots were stored at –80 °C for <6 mo. Treatments were supplemented into the oocyte maturation medium immediately after thawing at a final concentration of 25 ng/mL. Control treatments (CTR) consisted of carrier only (0.001% [*w/v*] BSA in TCM-199).

### 2.3. Oocyte Maturation 

Progression to metaphase II (MII) was completed as described previously [17,18]. At 16 or 22 h after beginning IVM, oocytes were denuded by vortexing then fixed in 4% [*w/v*] paraformaldehyde in Dulbecco’s phosphate-buffered saline (DPBS) for 15 min at room temperature. Oocytes were permeabilized with 1% [*v/v*] Triton X-100 in DPBS for 1 h at room temperature and blocked in 10% [*v/v*] donkey serum for 1 h at room temperature. Oocytes were incubated with a mix of 5 µg/mL mouse-ɑ-tubulin antibody and 5 µg/mL mouse anti-β-tubulin antibody (Sigma-Aldrich) either at room temperature for 1 h or overnight at 4 °C, then they were rinsed and incubated in Alexa Fluor 488 donkey anti-mouse (3.3 µg/mL) at room temperature for 1 h, and finally in 4′,6-diamidino-2-phenylindole (DAPI) (1 µg/mL) for 5 min at room temperature. Oocytes were placed into imaging droplets (DPBS containing 0.1% [*w/v*] *Polyvinylpyrrolidone [PVP]*) and imaged using an ECHO Revolve Epifluorescence Microscope with associated software (version R4) that includes the Z-stack software module (ECHO, San Diego, CA, USA). Oocytes were determined to have undergone meiosis II (MII) by detecting one polar body and a closely aligned cytoplasmic chromatin spindle. 

### 2.4. Real-Time Quantitative RT-PCR 

Total RNA was isolated from groups of COCs (n = 20/sample) after 4 or 22 h of IVM using the PureLink™ RNA Mini Kit. Adequate sample quality was verified (A_260/280_ > 1.8). Samples with adequate quality (A_260/280_ > 1.8; 5 ng) were treated with RNase-free DNase I at 37 °C for 30 min, the enzyme was inactivated (75 °C for 10 min), and then reverse transcription (RT) was completed using the High-Capacity cDNA Reverse Transcription Kit (Applied Biosystems, Foster City, CA, USA). The Fast SYBR^TM^ Green PCR Master Mix (Applied Biosystems) and primers (500 nM each; Table 1) were combined with RT products, and PCR was completed using the Applied Biosystems 7500 Fast PCR System (Applied Biosystems). Each sample was run in triplicate with an initial denaturation at 95 °C for 20 s followed by 40 cycles of 95 °C for 3 s and 60 °C for 30 s. Quantification of each transcript was calculated relative to the reference transcript, *HPRT1*. The reference transcript was found by others to serve as a suitable reference transcript for oocytes and embryos [19], and *HPRT1* abundance was not influenced by IVM culture time or cytokine treatment in our preliminary studies. Abundance relative to *HPRT1* was analyzed using the 2^−ΔΔCT^ method. 

### 2.5. In Vitro Fertilization and Culture

In vitro fertilization and embryo culture procedures were completed as previously described [16]. After 22 h IVM, COCs were washed and placed in groups of 25–30 in 500 µL fertilization medium covered with paraffin oil (Origio; Malov, Denmark). A mixture of frozen semen from four Holstein bulls (donation form Select Sires, Plain City, OH, USA) was thawed, and spermatozoa were isolated through a biphasic (40 and 80%, [*v/v*]) Bovipure ™ density gradient (Nidacon; Spectrum Technologies Healdsburg, CA, USA) before addition to the fertilization medium (1 million sperm/mL). Day 0 indicates the day of fertilization. After 14–18 h incubation with spermatozoa at 38.5 °C in 5% CO_2_ in humidified air, presumptive zygotes were denuded of cumulus cells, washed, and placed in groups of 20–30 in droplets of 50 µL of Synthetic Oviductal Fluid-Bovine Embryo 1 (SOF-BE1) medium [23] covered by paraffin oil. Zygotes were incubated at 38.5 °C in 5% O_2_, 5% CO_2_, 90% N_2_ in humidified air. 

Cleavage was assessed 72 h after IVF, and blastocyst development (out of total zygotes recovered from maturation and fertilization) was determined 7 and 8 days after IVF. Blastocyst stage was recorded as either regular (blastocyst with no increase in diameter relative to morulae), expanded (noteworthy increase in diameter and zona thinning), and hatched (release from the zona; blastocysts in the process of hatching were recorded in this category). The term “advanced blastocysts” included blastocysts categorized as either expanded or hatched. 

### 2.6. Embryo Cryopreservation and Thawing 

Day 8 regular and expanded blastocysts were cryopreserved according to previously published procedures with minor modifications [24,25]. Blastocysts were washed three times in HEPES-SOF then placed in a 1.5 M ethylene glycol solution containing 0.1 M sucrose (ViGRO Ethylene Glycol Freeze Plus; Vetoquinol Inc., Princeville, QC, Canada). Embryos were allowed to equilibrate for 5 to 15 min, until the embryos sank to the bottom of the dish, then were loaded into embryo transfer straws (5 blastocysts/straw) and sealed with a plastic plug. Straws were placed into the programmable slow freezer (Crysalys PTC 9500; Biogenics, Harriman, TN, USA) and held at −6 °C for 2 min. Straws were seeded using a liquid nitrogen covered cotton swab on the upper column of freezing media adjacent to the embryos and maintained at −6 °C for another 8 min before slow freezing at a rate of −0.6 °C/min. Upon reaching −32 °C, straws were plunged and stored in liquid nitrogen until subsequent thawing. 

Embryos were thawed using a Cito Thaw (CITO Products, Watertown, WI, USA) at 35.0 °C for 30 s. Embryos were expelled into HEPES-SOF and then were passed through three HEPES-SOF washes followed by two washes of SOF-BE1. Blastocysts were plated into 500 µL SOF-BE1 containing 10% [*w/v*] fetal bovine serum (FBS) (5 blastocysts/drop) that were covered in mineral oil and incubated at 38.5 °C in 5% CO_2_, 5% O_2_, and 90% N_2_ in humidified air. Blastocyst re-expansion, hatching, and degeneration were recorded at 24 and 48 h post-thawing. Re-expansion was determined by the re-emergence of a blastocoel cavity. Hatching was classified as detecting the re-expanded blastocyst either undergoing or completing zona hatching. Degenerate embryos represented those embryos that did not re-expand. 

### 2.7. Statistical Analyses

Least-squares analysis of variance (ANOVA) was completed using the general linear model (GLM) of the Statistical Analysis System (SAS; version 9.4; SAS Institute Inc., Cary, NC, USA). Pairwise comparisons were completed by using the probability of difference (PDIFF) test of SAS. Relative mRNA abundance data were log-transformed, and percentage data (i.e., oocyte, cleavage, and blastocyst data) were arcsine-transformed before analysis. Study replicate was used as the experimental unit for all analyses. All data are presented as non-transformed data. Statistical significance was determined at *p*  ≤  0.05 and a tendency in statistical significance was defined at *p* ≤ 0.1 and >0.05.

## 3. Results

### 3.1. Bovine COC Expression of IL6, IL11, LIF, and Their Receptors 

A recently published transcriptomic dataset [26] from bovine cumulus cells and denuded oocytes harvested immediately after COC retrieval from slaughterhouse-derived ovaries (i.e., not matured) was used to examine the presence or absence of expression for *IL6*, *IL11*, and *LIF,* and their receptors (Table 2). No *IL6*, *IL11*, or *LIF* transcripts were detected in the oocyte and cumulus cells, but transcripts for their receptors was detected. Both the cumulus cells and oocytes contained transcripts for *IL6ST* and *LIFR*, whereas transcripts for *IL6R* and *IL11RA* were detected in the oocyte but not the cumulus cells.

### 3.2. Lack of Cytokine Effect on Oocyte Maturation

**Figure 1:**
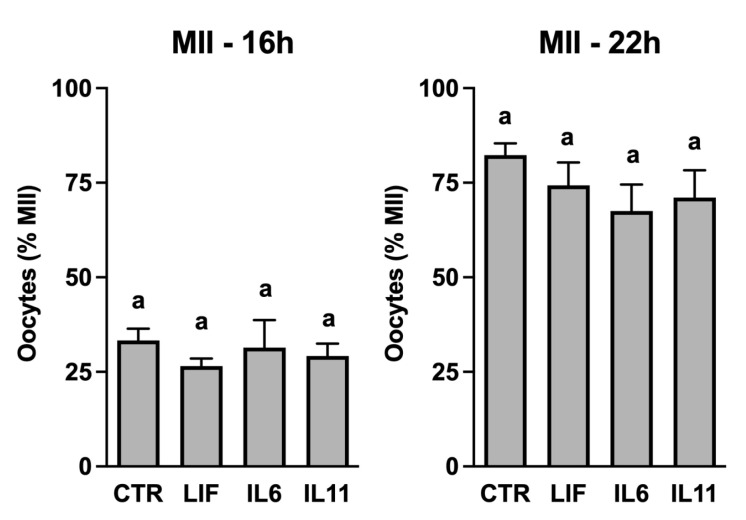
The progression of oocytes to metaphase II is not affected by supplementation with LIF, IL6, or IL11. Cumulus–oocyte complexes were matured in vitro in the presence of 25 ng/mL rhLIF, rhIL6, or rhIL11. Oocytes were harvested, denuded of cumulus cells, and processed for detection of first polar body extrusion and chromatin alignment after 16 h (left side graph) or 22 h (right side graph) (94–119 oocytes/treatment over six replicates). Different superscripts within each panel represent differences (*p* < 0.05).

The presence of IL6, IL11, and LIF receptor expression inspired us to explore whether these cytokines influence the rate of in vitro oocyte maturation (Figure 1). Maturation was assessed by examining the progression to the MII stage after 16 h (early maturation) and 22 h (normal duration of IVM). None of the cytokines influenced MII progression at either timepoint when provided at a concentration of 25 ng/mL. 

### 3.3. Effects of Cytokine Supplementation on Gene Expression

**Figure 2:**
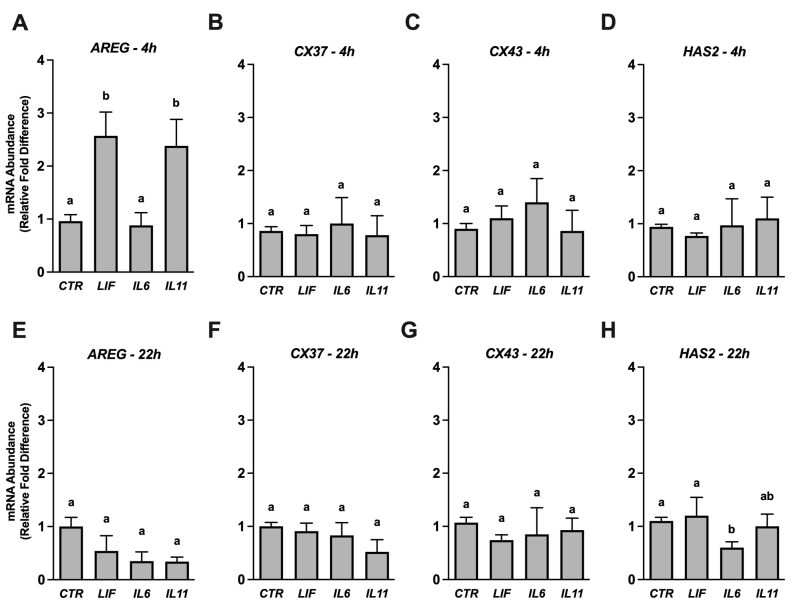
Effect of LIF, IL6, or IL11 supplementation on selective transcript abundances in COCs. Cumulus oocyte complexes were exposed to either no treatment (CONT) or to 25 ng/mL of either rhLIF, rhIL6, or rhIL11 during in vitro maturation, then COCs were harvested either 4 h (Panels (**A**–**D**); *n* = 4 replicate studies) or 22 h (Panels (**E**–**H**); *n* = 3replicate studies) later and processed to extract RNA and complete qRT-PCR. Shown are transcript abundances for *AREG* (Panels (**A**,**E**)), *CX37* (Panels (**B**,**F**)), *CX43* (Panels (**C**,**G**)), and *HAS2* (Panels (**D**,**H**)) relative to an internal control transcript (*HPRT1*) that are expressed as a fold-difference relative to the CTR value for each transcript. Different superscripts within each graph represent differences (*p* < 0.05).

Changes in the relative abundance of four transcripts described by others to serve as biomarkers of bovine cumulus cell competency [27,28,29,30] were used to explore whether IL6, IL11, or LIF influence cumulus cell competency during IVM (Figure 2). Transcript abundances were examined 4 h after beginning IVM and at the end of IVM (22 h). After 4 h of maturation, an increased abundance (*p* < 0.05) of *AREG* was detected with exposure to 25 ng/mL LIF or IL11 but not IL6 (Figure 2A). The expression of *CX37*, *CX43*, and *HAS2* was not affected by any treatment at the 4 h time-point (Figure 2B–D). After 22 h of maturation, no increases in transcript abundance were detected for any cytokine treatment (Figure 2E,H), but a reduction (*p* < 0.05) in HAS2 abundance was detected after IL6 treatment (Figure 2H). This effect was not generated after LIF or IL11 supplementation. 

### 3.4. Effects of Cytokine Supplementation on Subsequent Embryo Development 

**Figure 3:**
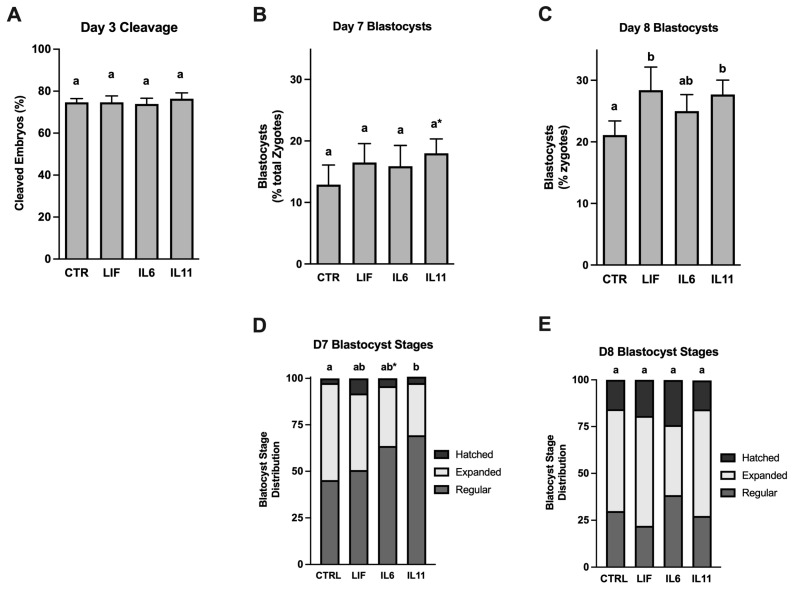
Effects of LIF, IL6, and IL11 supplementation during oocyte maturation on subsequent cleavage rate and blastocyst development at days 3, 7 and 8 post-fertilization. Cumulus–oocyte complexes were exposed to either no treatment (CONT) or to 25 ng/mL of either rhLIF, rhIL6, or rhIL11 during in vitro maturation, then treatments ended, and oocytes were fertilized and cultured to day 8 (*n* = 50–75 COCs/treatment/replicate; eight replicate studies). Shown here are the percentage of putative zygotes that cleaved by day 3 post-fertilization (Panel (**A**)) and that achieved the blastocyst stage by day 7 (Panel (**B**)) or day 8 (Panel (**C**)). Also shown are the distributions of regular, expanded, and hatched blastocysts on day 7 (Panel (**D**)) and day 8 (Panel (**E**)). Different superscripts within each graph represent differences (*p* < 0.05). The asterisks (*) indicates a tendency between the treatment group and the control group (*p* = 0.07).

A study was completed to examine whether supplementing IL6, IL11, or LIF during IVM will influence subsequent fertilization and in vitro embryo culture (Figure 3). None of the treatments affected cleavage rates (Figure 3A). At day 7 post-IVF, a tendency for an increase in blastocyst development (*p* = 0.07) in embryos derived from IL11-treated oocytes when compared with the control (Figure 3B). No effects of LIF or IL6 were detected on day 7. At day 8 post-IVF, blastocyst development was increased (*p* < 0.5) in embryos derived from LIF- and IL11-supplemented oocytes whereas IL6-supplemented oocytes produced blastocysts at the same frequency as the controls (Figure 3C). The distribution of blastocyst stages was different among the treatments. When compared with the controls, embryos derived from IL11-supplemented oocytes exhibited a reduction (*p* < 0.05) and those from IL6-supplemented oocytes tended to have fewer blastocysts (*p* = 0.07) that were expanded or hatched (Figure 3D). No treatment-dependent differences in the distribution in blastocyst stages were detected on day 8 (Figure 3E). 

### 3.5. Effects of Cytokine Supplementation on Embryo Cryopreservation Efficiency

**Figure 4:**
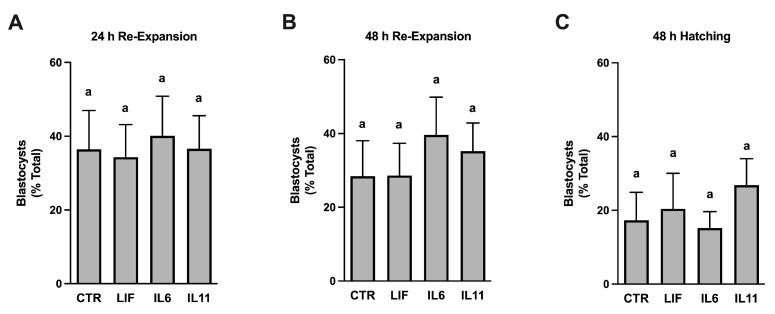
Post-thaw survival of cryopreserved bovine blastocysts after supplementation of LIF, IL6, or IL11 during oocyte maturation. Cumulus–oocyte complexes were exposed to either no treatment (CONT) or to 25 ng/mL of either rhLIF, rhIL6, or rhIL11 during in vitro maturation 8 (*n* = 5–15 blastocysts/treatment/replicated; eight replicate studies). Blastocysts were cryopreserved then thawed and incubated for 48 h to assess blastocyst re-expansion at 24 h (Panel (**A**)) and 48 h (Panel (**B**)) and hatching from the zona pellucida at 48 h (Panel (**C**)). Different superscripts within each graph represent differences (*p* < 0.05).

Work by others indicated that oocyte supplementation with a growth factor and cytokine cocktail containing LIF improves cryopreservation potential of bovine embryos [14]. A final study was, therefore, completed to determine if any of the cytokines studied in this work could act independently to improve bovine IVF blastocyst cryosurvival (Figure 4). The embryos produced from cytokine-treated oocytes contained the same ability as the controls to re-expand and hatch from the zona pellucida after 24 and 48-h. 

## 4. Discussion

The IL6 cytokine family are comprised of group of pleiotropic factors that act on various cells and tissues as differentiation, proliferation, apoptosis, and both pro- and anti-inflammatory factors [31,32]. Our interest in this cytokine family emerged from discovering that IL6 supplementation modifies the cellular composition of IVP bovine embryos to where they have a larger inner cell mass (ICM) and a greater number of primitive endoderm cells (PrE) [33]. The increased ICM size is interesting because bovine IVP blastocysts have fewer total ICM cells than their in vivo-produced counterparts [34,35,36,37], and this is potentially one reason why IVP embryos are less able to maintain viable pregnancies. The PrE is of interest because of its role in yolk sac development, and the observation that a subset of IVP conceptuses contain microscopic anomalies in yolk sac villous formation and vascular development [38,39]. These IL6 activities cannot be fully replicated by LIF supplementation [40], and detecting differential responses to different IL6 family members led to our speculation that the bovine oocyte may also respond differently to different IL6 family members. 

We included IL6, IL11, and LIF in these studies for two reasons. First, we verified that bovine COCs contain the receptor expression profiles necessary to respond to IL6, IL11, and LIF [41] (see Table 2). Transcripts for each of the three ligand-specific receptor subunits and the common, signaling transducing subunit (*IL6ST*) were present in cumulus cells. Only the LIF-specific receptor subunit (*LIFR*) and *IL6ST* were present within the oocyte, suggesting that LIF may contain activities that cannot be fully replicated by the other cytokines. Second, work completed by others determined that all three cytokines contained gene expression regulatory activity in bovine COCs [13]. This previous work also found that each cytokine functioned at or below the concentration we tested (25 ng/mL). There is an absence of *IL6*, *IL11*, and *LIF* mRNA in both the cumulus cells and oocyte immediately after COCs are harvested, but dynamic changes in the gene expression of *IL6* and *LIF* exist during bovine oocyte maturation. Specifically, both *IL6* and *LIF* mRNA peaks 6 to 7 h after the resumption of meiosis occurs in bovine COCs [13,15]. To our knowledge, the expression profile for *IL11* has not been examined. 

The interest in exploring whether these cytokines influence the ontogeny and overall efficiency of meiotic progression to MII stemmed from previous work where *IL6* and *IL6ST* expression profiles in bovine COCs were shifted 2 h earlier upon exposure to elevated incubation temperature [15]. This finding is noteworthy because COC exposed to this heat shock condition matured sooner than the thermoneutral controls [42]. This led us to propose that IL6 may be responsible for this rapid completion in progression to MII. This was not observed. No differences in MII progression were detected at 16 h post-IVM. Rather, a reduction in MII efficiency was detected in the IL6-supplemented COCs at 22 h post-IVM. This reduction did not have an adverse effect on cleavage rate and blastocyst development, so it is unclear whether this outcome is biologically important. 

The expression profiling study utilized previously validated cumulus markers of COC competency to describe whether any of the cytokines under investigation affect cumulus cell activity during IVM [20,29,43]. Protein products for *CX37* and *CX43* play pivotal roles in controlling granulosa and cumulus–oocyte gap-junctional communication [44], *HAS2* is involved with cumulus expansion [45], and *AREG* transduces gonadotrophin effects within the COC [46]. Both LIF and IL11 increased *AREG* transcript abundance at 4 h. This may indicate that these cytokines can enhance the effects of LH and FSH during maturation [47]. Supplementation with IL6 did not influence *AREG* transcript abundance, but it reduced *HAS2* transcript abundance after 22 h. Although cumulus expansion was not examined in this work, it is interesting that fewer oocytes supplemented with IL6 progressed to MII by 22 h. Perhaps these two adverse events are related to one another. It certainly is possible that IL6 is not able to target the same signaling systems as LIF and IL11 within the COC. Further work is needed to describe these and other ligand-specific activities. 

Arguably, the most notable outcome of this study was observing LIF and IL11-dependent improvements in blastocyst development at day 8 post-IVF. The IL11 finding has not been described previously. Positive effect of LIF on blastocyst rates has been observed in cattle in some studies [12] but not in others [14]. The positive effect of LIF and IL11 on blastocyst development was not dependent on the effects of these supplements on fertilization success. No beneficial effects of cytokine supplementation during IVM were detected on cleavage rate. This and other post-maturation effects have not been explored for IL6 for IL11, but there are several reports showing improvements in cleavage rates after LIF supplementation in cattle, pigs, goats, and mouse COCs [12,48,49,50,51]. 

It is difficult to develop solid reasons for the absence of beneficial effects of IL6 supplementation on blastocyst development. No adverse effects on cleavage rate were detected in this treatment group, but there was a statistical tendency for a reduction in the proportion of advanced and hatched blastocysts in this group at day 7 post-IVF. This effect was not detected on day 8, and it is tempting to speculate that embryos from IL6 and IL11-supplemented oocytes are developing slower than embryos supplemented with LIF. This finding is intriguing because others have claimed that the fastest developing IVP embryos are not necessarily the most competent embryos. Embryos developing at a slower rate will have a “quiet” metabolism that is conducive to optimize post-transfer competency [52]. Recently, this “Quiet Embryo Hypothesis” has been refined to consider that having too low of a metabolic rate is detrimental. Having an optimal median range in metabolic activity, termed the “Goldilocks Zone”, appears to maximize developmental competency of IVP embryos [53]. 

We are confident that the recombinant IL6 protein contains biological activity. Our laboratory recently transitioned away from using a largely uncharacterized bovine recombinant protein to the human recombinant IL6 protein used in this work that has been thoroughly evaluated for purity and biological activity. We consistently receive positive effects on bovine embryos at a concentration of 20–25 ng/mL (Oliver and Ealy, unpublished observations). There is a remote possibility that a different concentration of human recombinant IL6 is needed to observe a more robust effect on bovine blastocyst development. Many growth factors and cytokines contain biphasic, dose-dependent effects when used for in vitro work. However, another group determined that microRNA expression was increased when using a similar concentration of human recombinant IL6 [13]. One final possibility is that sufficient COC-derived IL6 is being produced during IVM, whereby supplemented IL6 was not needed to provide the maximal benefit of this cytokine. Although we failed to detect IL6 mRNA at the beginning of maturation, another laboratory discovered that IL6 mRNA levels increase at 4 to 8 h after the beginning of maturation [15]. No reports exist that have quantified IL6 protein abundances in bovine COCs, but IL6 protein has been detected in the mouse COC, and this COC-derived IL6 plays an active role in mediating cumulus expansion [54]. 

The final study explored whether cytokine treatment during IVM improves subsequent embryo cryosurvival. This work was inspired by a study completed by Stoecklein and colleagues [14], where they found that supplementing LIF along with FGF2 and IGF1 during IVM improved bovine blastocyst survival following slow freezing. No such beneficial effects were observed when supplementing LIF, IL6, or IL11. These disparate outcomes indicate that mixing LIF and potentially also IL6 or IL11 with other factors is likely necessary to improve bovine IVP embryo cryosurvival, or this outcome may reflect differences in culture systems, cryopreservation protocols, and/or post-thaw analyses that may exist between this and studies completed by other laboratories. 

## 5. Conclusions

These findings have led us to propose that supplementing either LIF or IL11 during IVM may be beneficial to IVP embryo development. The mechanism(s) of action for the improvements in IVP blastocyst yields have not been defined, but observing positive effects of either cytokine on selective COC transcripts is consistent with the hypothesis that LIF and IL11 alter COC gene expression in ways that promote subsequent embryo development. Further work is needed to define specific pathways and biological systems that are controlled by these cytokines. By contrast, IL6 supplementation did not produce positive effects on COC gene expression, oocyte maturation, and blastocyst development. Possible explanations for these outcomes are that sufficient endogenous IL6 production occurs during IVM, so further supplementation is not needed. 

## Figures and Tables

**Table 1:** Primer sequences used for qRT-PCR.

Transcript	Primer Sequence *	Reference
*AREG*	F: 5′-CTTTCGTCTCTGCCATGACCTT-3′	[20]
	R: 5′-CGTTCTTCAGCGACACCTTCA-3′	
*CX37*	F: 5′-GACTCATCTCCCTGGTGCTC-3′	[21]
	R: 5′-GTTCTGCTCACTGGACGACA-3′	
*CX43*	F: 5′-GTCTTCGAGGTGGCCTTCTTG-3′	[21]
	R: 5′-AGTCCACCTGATGTGGGCAG-3′	
*HAS2*	F: 5′-GGGTTCTTCCCTTTCTTTCT-3′	[22]
	R: 5′-CCACCCAGCTTTGTTTATTG-3′	
*HPRT1*	F: 5′-TGCTGAGGATTTGGAGAAGG-3′	[19]
	R: 5′-CAACAGGTCGGCAAAGAACT-3′	

* F: forward, 5′ primer; R: reverse, 3′ primer.

**Table 2:** Expression profiles of IL6, IL11, and LIF and their receptor subunits in bovine oocyte and cumulus cells harvested before in vitro maturation.

	Oocyte (Mean FPKM *)	Cumulus (Mean FPKM *)
**Ligand**		
*IL6*	ND	ND
*IL11*	ND	ND
*LIF*	ND	ND
**Receptor**		
*IL6ST*	2.57	17.37
*IL6R*	ND	9.27
*IL11R*	ND	5.82
*LIFR*	8.00	11.89

* FPKM: Fragments per kilobase per million reads; ND: Not Detected Adapted from [26].

## Data Availability

All data collected in this work are available for viewing upon submission of a reasonable request to the corresponding author at ealy@vt.edu.
